# Mapping the Scientific Landscape of Schizophrenia Genetics (2020–2025): A Bibliometric and Scientometric Study of Global Trends and Collaborations

**DOI:** 10.1002/brb3.71171

**Published:** 2025-12-31

**Authors:** Niloofar Mikaeili, Mahdi Naeim, Mohammad Narimani

**Affiliations:** ^1^ Department of Psychology, Faculty of Educational Sciences and Psychology University of Mohaghegh Ardabili Ardabil Iran

**Keywords:** bibliometrics, GWAS, genetics, schizophrenia, VOSviewer

## Abstract

**Background and objective:**

This study provides a comprehensive bibliometric and scientometric analysis of research trends in schizophrenia genetics from 2020 to 2025. Driven by rapid technological advances such as genome‐wide association studies (GWAS) and next‐generation sequencing (NGS), the field has experienced significant growth. The objective was to map the thematic evolution, key contributors, and collaboration networks shaping psychiatric genomics research.

**Methods:**

An integrated bibliometric and scientometric approach was employed. A dataset of 5001 English‐language publications authored by 27,692 researchers was retrieved from PubMed, Scopus, and web of science (Wos) databases. Following deduplication and eligibility screening, VOSviewer (version 1.6.20) was used to generate network visualizations analyzing co‐authorship, keyword co‐occurrence, citation, and co‐citation patterns.

**Results:**

The analysis revealed a notable surge in publications after 2022, increasing from 431 articles in 2020 to 1,645 articles in 2022, before stabilizing above 900 publications annually in 2023 and 2024. Six major thematic clusters were identified: (1) genetic and cellular mechanisms; (2) neurochemical and behavioral studies; (3) neuroimaging; (4) clinical and epidemiological features including polygenic risk scoring; (5) methodological approaches; and (6) comorbidities with pharmacogenetic implications. Prominent contributors included Ole A. Andreassen and Vince D. Calhoun, while the United States, China, and the United Kingdom emerged as central hubs of international collaboration.

**Conclusion:**

This study elucidates the leading researchers, prevailing themes, and collaborative networks driving schizophrenia genetics research from 2020 to 2025. The findings demonstrate the efficacy of bibliometric and scientometric methods, particularly VOSviewer, in revealing the dynamic landscape of psychiatric genomics and guiding future multidisciplinary research efforts.This graphical abstract illustrates the integrated workflow of the study “mapping the scientific landscape of schizophrenia genetics (2020–2025)”. The process combines bibliometric and scientometric analyses of 5,001 publications using VOSviewer to identify thematic clusters, key contributors, and international collaboration networks that define global research trends in schizophrenia genetics.

## Graphical abstract

This graphical abstract illustrates the integrated workflow of the study‐ Mmapping the Sscientific Llandscape of Sschizophrenia Ggenetics (2020‐2025). The process combines bibliometric and scientometric analyses of 5,001 publications using VOSviewer to identify thematic clusters, key contributors, and international collaboration networks that define global research trends in schizophrenia genetics.

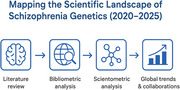

## 1 Introduction

Schizophrenia is one of the most complex and debilitating psychiatric disorders, affecting approximately 0.3% to 1% of the global population (Tandon et al. 2024; Stefansson et al. 2009; Sullivan et al. 2012). Characterized by hallucinations, delusions, disorganized thinking, and cognitive impairments, it profoundly impacts individuals, families, and society (Tandon et al. 2024). In 2019, the global burden of schizophrenia was estimated at 23.6 million cases, accounting for 12.66 million disability‐adjusted life years (DALYs) (Velligan and Rao, 2023; Safiri et al. 2024). Beyond its chronic and disabling nature, the disorder imposes significant economic costs due to direct medical expenses and indirect losses such as reduced productivity (Solmi et al. 2023). Genetic, environmental, and neurophysiological factors contribute to its etiology, highlighting the need for extensive research to unravel its complexity and develop effective treatments (McGrath et al. 2008).

Genetic research over recent decades has greatly advanced our understanding of schizophrenia's biological underpinnings. GWAS have confirmed the polygenic nature of schizophrenia, with numerous common variants contributing small effects (Owen et al. 2023; Legge et al. 2021). For example, large‐scale GWAS have identified risk loci such as the major histocompatibility complex (MHC) region, while exome sequencing has revealed rare variants in genes like SETD1A, which confer substantial risk (Legge et al. 2021). Key susceptibility genes such as NRG1 and DISC1 have also been implicated, alongside overlaps with bipolar disorder and other neurodevelopmental conditions (Merikangas et al. 2022; Dennison et al. 2020). While these findings pave the way for precision medicine, the underlying genetic complexity still poses translational challenges (Van de Leemput et al. 2016).

Bibliometric analysis is a quantitative method for mapping scientific literature, identifying key research themes, trends, and collaboration networks (Jin et al. 2025). Tools like VOSviewer enable visualization of co‐authorship networks, keyword co‐occurrence, and citation patterns (Canul‐Medina et al. 2024). For example, Jin et al. (2025) used VOSviewer to map biomarker research in schizophrenia, revealing clusters around “genetics,” “proteomics,” and genes like NRXN1 and CNTNAP2. Another study explored the global landscape of schizophrenia and serotonin research, highlighting genetic concepts such as “pharmacogenetics” and “epigenetics” (Canul‐Medina et al. 2024).

Given the biological complexity and public health impact of schizophrenia, innovative approaches are needed to improve understanding and management. Bibliometric analysis of genetic research using VOSviewer offers a novel and comprehensive overview of the field. This approach helps uncover research hotspots, collaboration patterns, and emerging gaps, informing future scientific and clinical directions (Canul‐Medina et al. 2024). For instance, identifying genetic biomarkers such as DNA methylation changes could support more accurate diagnoses and personalized treatments (Jin et al. 2025). Strengthening international collaborations, especially in underrepresented regions like the Middle East, may further accelerate progress (Safiri et al. 2024).

This study aims to conduct a comprehensive bibliometric analysis of schizophrenia genetics research. It identifies key and emerging themes, maps collaboration networks among authors, institutions, and countries, and explores temporal and thematic trends to highlight research trajectories and knowledge gaps. Ultimately, it seeks to provide an integrated perspective that supports future investigation and clinical translation in the field.

## 2 Literature Review

Bibliometric analyses serve as an essential tool for mapping the research landscape in schizophrenia and other psychiatric disorders, especially through the use of software such as VOSviewer. These studies allow for the identification of research trends, prominent authors, and patterns of international collaboration. For example, Jin et al. (2025) conducted a comprehensive bibliometric analysis of the literature on diagnostic biomarkers in schizophrenia. By employing VOSviewer and CiteSpace, they identified thematic clusters related to genetics, including terms like “genetics” and “gene expression,” which emphasized the role of GWAS and DNA methylation changes (Jin et al. 2025). In a similar vein, Canul‐Medina et al. (2024) used VOSviewer to examine global research on the connection between schizophrenia and serotonin, highlighting areas such as pharmacogenomics and epigenetics. This demonstrates the widespread application of this tool in psychiatric research (Canul‐Medina et al. 2024). Another study by Fu et al. (2024) utilized VOSviewer to analyze research related to resting‐state functional magnetic resonance imaging (fMRI) in schizophrenia and identified international collaborations and temporal trends (Fu et al. 2024). These findings underscore the ability of VOSviewer to visualize complex scientific networks such as co‐authorship and keyword co‐occurrence in medical and neuroscience research.

The use of VOSviewer in bibliometric analyses extends beyond the field of schizophrenia and is prominent in various areas of medicine and neuroscience. For instance, Sun et al. (2022) employed this software to explore research concerning inflammation in schizophrenia and visualized collaboration networks among countries (Sun et al. 2022). Additionally, in non‐psychiatric domains such as e‐learning, VOSviewer has been applied to analyze keyword co‐occurrence and identify research trends (Martins et al. 2024). The tool creates visual maps based on item proximity, where the closeness between elements indicates the strength of their relationship, thus enabling detailed analysis of scientific connections (Van Eck and Waltman 2010). In neuroscience, VOSviewer has been used to identify research clusters in fields such as brain imaging and biomarkers, illustrating its adaptability in handling complex data structures (Chen et al. 2012).

Despite these advancements, there are still notable gaps in bibliometric analyses focusing specifically on schizophrenia genetics. Zakaria et al. (2023) conducted a bibliometric analysis of schizophrenia genetic research before and after the introduction of GWAS and observed a shift toward precision medicine. However, their study only covered research up to the year 2021 (Zakaria et al. 2023). Similarly, Jin et al. (2025) investigated genetic biomarkers, but their focus was on a broader range of biomarkers rather than genetics alone (Jin et al. 2025). In light of rapid developments in genomic technologies such as whole genome and exome sequencing, and the growing number of genetic loci associated with schizophrenia, which now exceed 270 according to recent reports (Legge et al. 2021), there is a clear need for updated bibliometric analyses specifically focused on schizophrenia genetics. Furthermore, a shortage of bibliometric studies addressing non‐European populations and the clinical applications of genetics, including pharmacogenomics and gene–environment interactions, represents another important research gap (Dennison et al. 2020). These shortcomings create opportunities for future investigations to provide a more comprehensive understanding of genetic research in schizophrenia.

Overall, existing bibliometric studies have laid the groundwork for understanding research trends in schizophrenia. However, there remains a strong need for more focused and current studies in the field of schizophrenia genetics. This need is especially relevant in light of recent genomic advancements and the growing imperative to translate genetic findings into clinical applications. Such studies could help identify leading researchers, top institutions, and emerging directions in the field and ultimately contribute to improving personalized treatment approaches for schizophrenia.

## 3 Materials and Methods

### 3.1 Data Sources

Thi**s** bibliometric and scientometric analysis was conducted using publication data retrieved from three major databases: PubMed, Scopus, and WoS. These databases were selected due to their comprehensive coverage of biomedical and psychiatric literature and their alignment with the indexing standards of reputable journals. The study focused on publications published between January 1, 2020, and July 6, 2025, a period marked by significant advancements in genomic technologies relevant to schizophrenia research, including GWAS and NGS.

### 3.2 Search Strategy

A structured search strategy was developed to identify relevant literature on schizophrenia genetics across three major databases: PubMed, Scopus, and WoS. The search combined key terms including “schizophrenia,” “genetics,” “genomics,” “GWAS,” “polygenic risk score,” “epigenetics,” and “pharmacogenomics,” using Boolean operators to maximize retrieval accuracy.

The PubMed query was formulated as follows:

(“schizophrenia”[Title/Abstract] AND (“genetics”[Title/Abstract] OR “genomics”[Title/Abstract] OR “GWAS”[Title/Abstract])) AND (“2020/01/01”[PDat] : “2025/07/06”[PDat]) AND English[Lang].

For Scopus, the search syntax was adapted to:

(TITLE‐ABS‐KEY(schizophrenia) AND (TITLE‐ABS‐KEY(genetics) OR TITLE‐ABS‐KEY(genomics) OR TITLE‐ABS‐KEY(GWAS))) AND (PUBYEAR > 2019 AND PUBYEAR < 2026) AND (LIMIT‐TO (LANGUAGE, “English”)).

Similarly, the WoS query was:

TS = (schizophrenia AND (genetics OR genomics OR GWAS)) AND PY = 2020–2025 AND LA = English.

All queries were limited to articles and reviews to exclude editorials, conference abstracts, and non‐English documents.

### 3.3 Inclusion and Exclusion Criteria

Publications were included if they were written in English, published between 2020 and 2025, and focused on schizophrenia in the context of genetic or genomic research, as identified through titles, abstracts, or keywords. Articles unrelated to genetics, such as purely clinical studies, as well as grey literature including editorials and conference proceedings, were excluded from the analysis.

### 3.4 Data Processing and Cleaning

A total of 5501 articles were initially retrieved from the combined searches of PubMed, Scopus, and WoS. After applying the inclusion and exclusion criteria and removing duplicate records, 5001 articles were retained and entered into the final analysis dataset. The bibliographic data were exported in CSV and RIS formats compatible with bibliometric analysis software. Prior to analysis, data cleaning and preprocessing were performed using Microsoft Excel and Python (pandas library). This process involved deduplication of records, keyword harmonization by merging synonyms (e.g., “polygenic risk scores (PRS)” and “polygenic risk score”) and unifying singular/plural forms, and validation of metadata accuracy through both automated scripts and manual curation to ensure data integrity.

### 3.5 Bibliometric Analysis and Visualization

Quantitative analysis and visualization of bibliometric networks were conducted using VOSviewer (version 1.6.20). This software was selected for its capabilities to construct and visualize co‐authorship, keyword co‐occurrence, citation, and co‐citation networks. The primary analyses included co‐authorship to examine collaborative structures among authors, institutions, and countries; keyword co‐occurrence to identify core research themes; and citation analysis to highlight influential publications and the intellectual framework of the field. Thresholds were set to enhance analytical clarity, including a minimum of ten occurrences for keyword co‐occurrence, at least five publications for author co‐authorship, and a minimum of ten publications for country‐level collaboration.

### 3.6 Network Mapping Procedure

The bibliometric mapping followed a systematic procedure beginning with data extraction from the three selected databases, followed by data cleaning and importing into VOSviewer. Parameters were adjusted for each type of analysis, and networks were generated using the software's distance‐based visualization and clustering algorithms. The resulting clusters were interpreted to identify thematic areas, key contributors, and patterns of collaboration within the schizophrenia genetics research community.

## 4 Results

### 4.1 Overview of Publications

Initially, a total of 5501 articles related to schizophrenia genetics were identified. After removing duplicates and applying inclusion and exclusion criteria, 5001 articles published between 2020 and 2025 were included in the analysis. These publications involved 27,692 unique authors, reflecting a large and active research community.

### 4.2 Annual Publication Trends

Table 1 demonstrates a marked escalation in annual publication output starting in 2022, jumping from fewer than 450 articles per year to over 1600. This sharp rise likely reflects a paradigm shift in research priorities, driven by expanded funding streams and the increasing accessibility of genomic methodologies such as GWAS and NGS. The continued high levels of publication in 2023 and 2024 suggest that this is not a transient spike but rather the emergence of a sustained research trajectory. The modest decrease observed in 2025 is more plausibly attributed to temporal delays in database indexing or publication processing rather than an actual decline in scientific activity.

**TABLE 1:** Presents the distribution of publications over time.

Year	Number of articles
2020	431
2021	424
2022	1645
2023	942
2024	1018
2025	540

*Note*: This table shows the annual number of publications included in the bibliometric analysis for the period 2020‐2025.

### 4.3 Leading Authors

Table 2 identifies the leading contributors in schizophrenia genetics research between 2020 and 2025, revealing a highly centralized authorship structure. Notably, Andreassen, Ole A. and Calhoun, Vince D. occupy central positions in the global co‐authorship network, reflecting both prolific output and strategic collaboration across high‐impact projects. Their repeated appearances in international consortia suggest institutional leadership and resource concentration, which may influence the thematic direction of the field. The clustering of authors such as Kochunov, Peter and Djurovic, Srdjan around these hubs further supports the presence of stable research alliances. This pattern implies that knowledge production in schizophrenia genetics is shaped by a relatively small set of influential figures, potentially affecting diversity in research perspectives and access to funding.

**TABLE 2:** Highlights the top contributing authors in the field between 2020 and 2025.

Author	Publications
Andreassen, Ole A	69
Calhoun, Vince D	54
Kochunov, Peter	30
Weinberger, Daniel R	26
Djurovic, Srdjan	25
Papiol, Sergi	24
Dannlowski, Udo	24
Turner, Jessica A	23
Van Os, Jim	23
Bearden, Carrie E	22

*Note*: This table lists the most productive authors between 2020 and 2025 based on the number of publications included in the bibliometric analysis.

### 4.4 International Collaboration and Institutional Contributions

Leading countries in schizophrenia genetics research include the United States, China, the United Kingdom, Germany, and Norway. Key contributing institutions are:

- University of Oslo
- National Institute of Mental Health (USA)
- King's College London
- University of Heidelberg

### 4.5 Network Analysis Using VOSviewer

A series of network maps was generated using **VOSviewer**, providing insight into co‐authorship, keyword co‐occurrence, citation, and co‐citation patterns.

**FIGURE 1:**
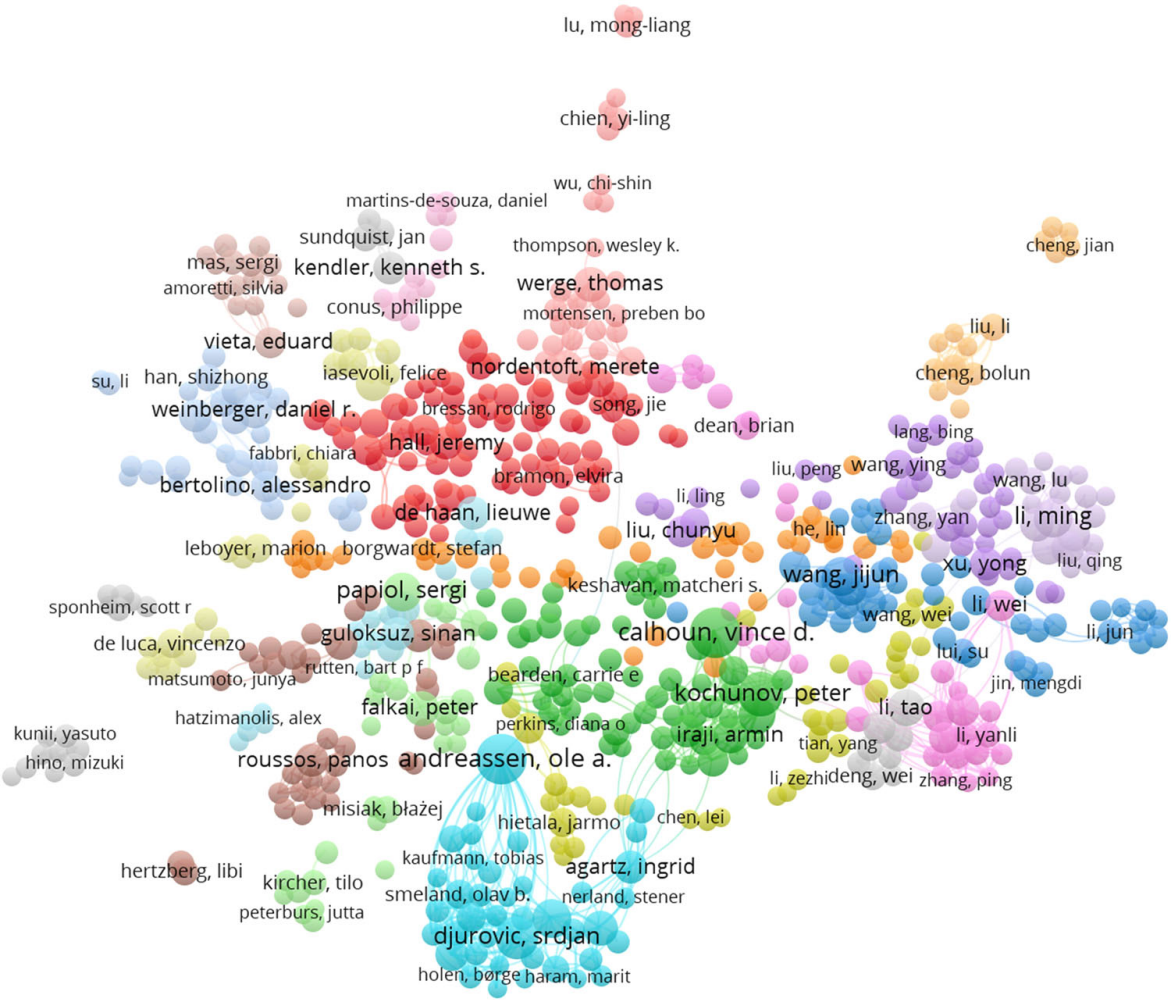
Co‐authorship network.

As illustrated in Figure 1, the co‐authorship network shows a highly centralized collaboration structure. Ole A. Andreassen (69 publications) and Vince D. Calhoun (54 publications) occupy central positions, reflecting their leadership in international consortia. Surrounding clusters, including collaborators such as Peter Kochunov and Daniel R. Weinberger, highlight the dominance of a few prolific groups in shaping schizophrenia genetics research.

**FIGURE 2:**
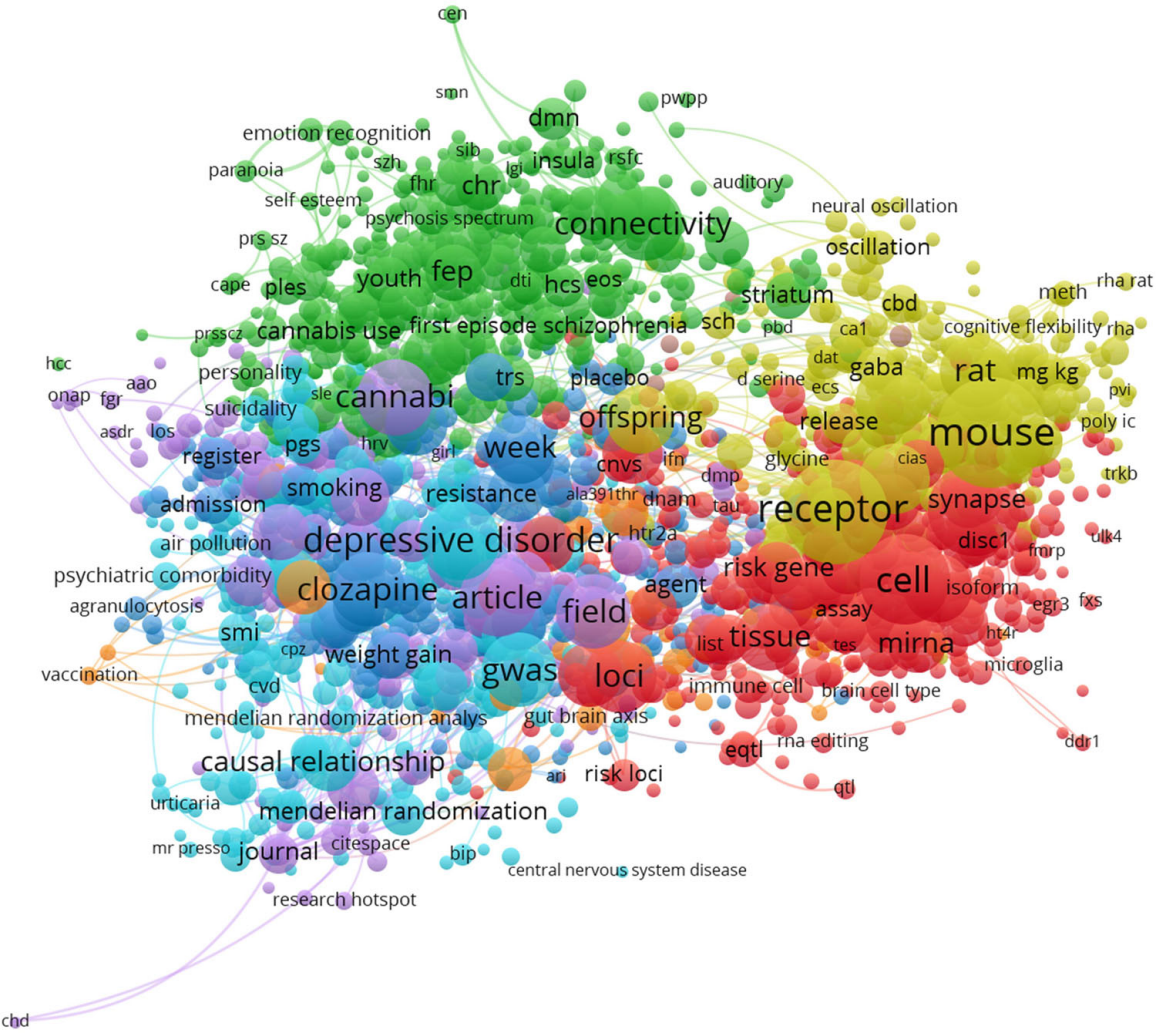
Keyword co‐occurrence network.

As illustrated in Figure 2, six major thematic clusters were identified: genetic and cellular mechanisms, neurochemical and behavioral studies, neuroimaging, clinical and epidemiological features (notably PRS and GWAS), methodological approaches, and comorbidities with pharmacogenetic implications. High‐frequency keywords such as GWAS, PRS, and DISC1 emphasize the integration of genetic risk with clinical and imaging studies.

**FIGURE 3:**
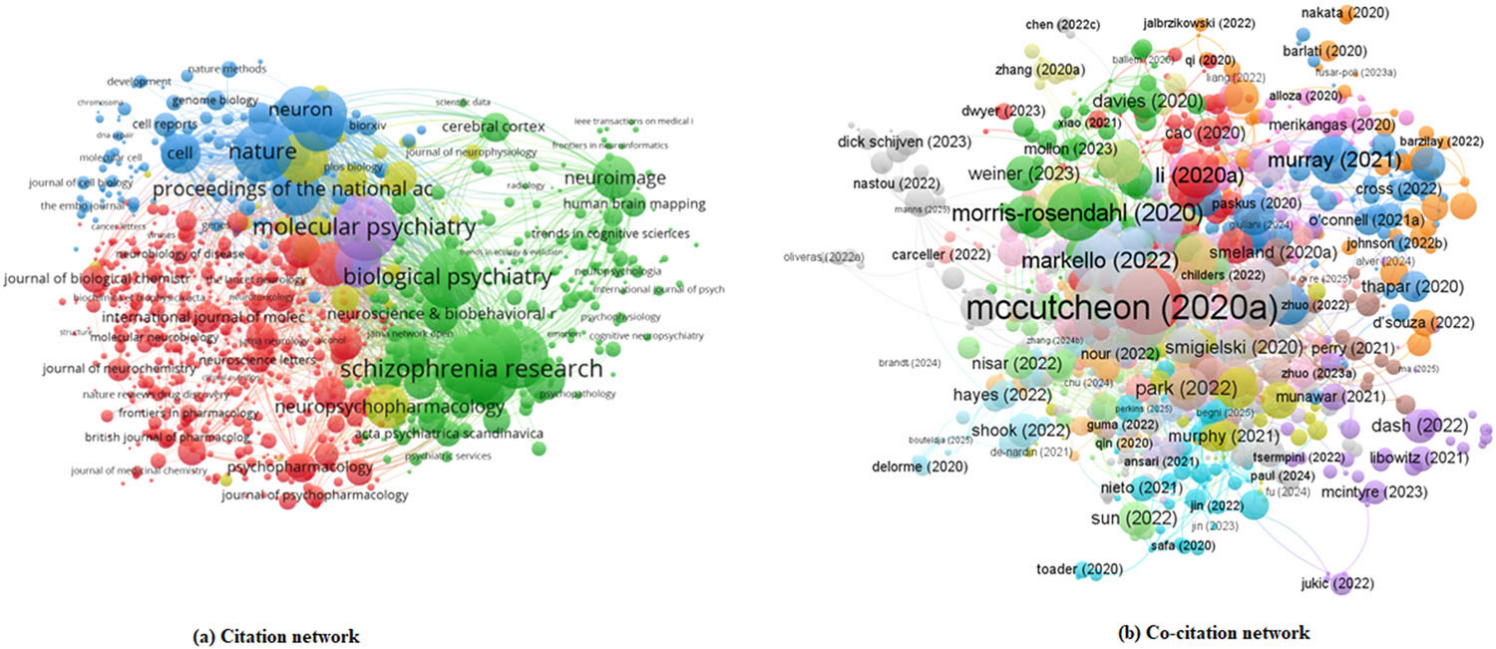
Citation and co‐citation networks in schizophrenia genetics (2020–2025).

As illustrated in Figure 3, the citation and co‐citation networks together map the intellectual structure of schizophrenia genetics research. In panel (a), the citation network highlights influential studies, with articles such as McCutcheon et al. (2020) and Smeland et al. (2020) emerging as central hubs. These works link genetic discoveries with neurobiological and clinical models, guiding subsequent research on PRS‐based stratification and neuroimaging integration. In panel (b), the co‐citation network emphasizes the journals that anchor the field, with schizophrenia research, biological psychiatry, and molecular psychiatry forming the psychiatric core, while nature, neuron, and cell represent connections to broader molecular and neuroscience domains. Together, these networks demonstrate how key publications and journals collectively shape the conceptual and methodological development of schizophrenia genetics.

**FIGURE 4:**
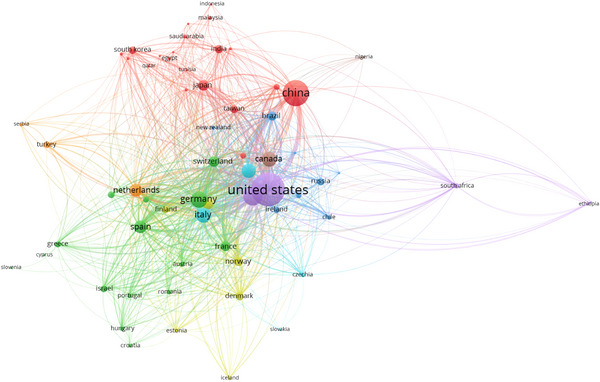
International collaboration network.

As illustrated in Figure 4, the United States stands as the central hub with over 1200 publications, forming strong collaborative ties with China and the United Kingdom. Other significant contributors include Germany and Norway, while regions such as the Middle East remain underrepresented, indicating persistent global disparities in research capacity.

## 5 Discussion

This bibliometric analysis highlights significant trends and patterns in schizophrenia genetics research between 2020 and 2025. The results underscore a sharp rise in publication volume beginning in 2022, increasing from fewer than 450 papers annually in 2020–2021 to more than 1600 in 2022 (see Table 1). This surge reflects not only the expanded application of GWAS and NGS but also the growing accessibility of large‐scale datasets such as biobank resources and international consortia, which have accelerated discovery and data sharing (Andreassen et al. 2014; Lee et al. 2019).

Thematic cluster analysis demonstrated that schizophrenia genetics is characterized by six interconnected domains rather than isolated lines of inquiry. GWAS and PRS provided quantitative estimates of genetic liability, which have increasingly been integrated into neuroimaging studies to examine structural and functional correlates of genetic risk (McCutcheon et al. 2020). For instance, PRS has been linked to alterations in brain connectivity and activation patterns, thereby bridging molecular variation with intermediate phenotypes (McCutcheon et al. 2020). Similarly, animal model and neurochemical research offered mechanistic support by validating the biological significance of candidate genes and neurotransmitter pathways, such as glutamatergic and GABAergic signaling, that emerged from GWAS findings (Malhotra and Sebat 2012; Dennison et al. 2020). Clinical and epidemiological studies have extended these insights by incorporating PRS into patient stratification models, demonstrating its potential utility in predicting disease onset, treatment response, and comorbidity risk (Zhang et al. 2019). Pharmacogenetic studies, in turn, are beginning to explore how genetic variation influences medication safety and efficacy, such as differential clozapine response, pointing toward a future of more personalized psychiatry (Smeland et al. 2020). Methodological advances, including Mendelian randomization and multi‐omic integration, provide the analytical backbone for linking these domains, allowing causal inference and cross‐validation across genetic, molecular, and clinical layers (Smeland et al. 2020; Legge et al. 2021). Together, these clusters illustrate a field moving toward translational integration, where molecular discoveries inform neural models, which in turn guide clinical practice.

The citation and co‐citation analyses emphasized the central role of foundational works such as McCutcheon et al. (2020) and Smeland et al. (2020), which have guided the integration of GWAS data with transcriptomic and imaging evidence. These hubs reflect the intellectual architecture of schizophrenia genetics, where influential studies continue to shape subsequent research agendas.

The international collaboration network confirmed that the United States remains the leading hub of schizophrenia genetics research, contributing over 1200 publications during the study period, followed closely by China, the United Kingdom, and Germany. This dominance is partly attributable to access to large‐scale biobank datasets and major funding initiatives (Andreassen et al. 2014; Lee et al. 2019). However, contributions from underrepresented regions such as the Middle East remain limited, despite growing disease burden in these populations (Safiri et al. 2024). Addressing this gap through greater inclusion of diverse genetic cohorts is essential to improve the generalizability of findings and reduce Eurocentric bias in psychiatric genomics.

Limitations of the present study should be acknowledged. While combining PubMed, Scopus, and WoS improved coverage, the exclusion of non‐English publications and grey literature likely introduced language and publication bias. Furthermore, the accuracy of bibliometric analysis depends heavily on metadata quality, which may obscure nuanced content. VOSviewer, though widely used, is sensitive to keyword synonymy and semantic variation; for example, terms such as “polygenic risk score” and “PRS” may be treated as distinct nodes unless rigorously standardized. To mitigate this, keywords and synonyms were standardized prior to analysis (e.g., “PRS” → “polygenic risk score”) through manual curation and Python scripts. In addition, emerging areas of research such as single‐cell genomics, spatial transcriptomics, and multi‐omic integration may be underrepresented due to their novelty and inconsistent indexing. Finally, while the analysis highlights central authors and countries, the dominance of prolific figures and resource‐rich nations risks overshadowing contributions from smaller groups and underrepresented regions.

Future research should address these gaps by incorporating additional data sources such as preprint servers and regional databases, applying advanced natural language processing tools to harmonize terminology, and explicitly analyzing the role of underrepresented populations in psychiatric genetics. Combining bibliometric mapping with systematic reviews and meta‐analyses would also provide a more comprehensive synthesis, particularly for translational domains such as pharmacogenomics and precision medicine.

## 6 Conclusion

This bibliometric analysis offers a comprehensive overview of global research trends in schizophrenia genetics from 2020 to 2025. The findings reveal a marked increase in publication activity beginning in 2022, likely reflecting the growing integration of advanced genomic tools such as GWAS and NGS in psychiatric genetics. Influential authors, including Andreassen, Calhoun, and Kochunov, emerged as central contributors, while the United States, China, and several European nations demonstrated strong collaborative networks. Thematic clustering of keywords identified six major research domains, ranging from molecular mechanisms and animal models to neuroimaging and comorbidities. Importantly, these domains are increasingly interlinked, for example, PRS derived from GWAS are now being applied in neuroimaging studies and clinical stratification, while pharmacogenetic research connects genetic variation to treatment outcomes. The citation and co‐citation networks highlighted seminal works and interdisciplinary knowledge flows, particularly between psychiatry, molecular biology, and neuroscience. These insights underscore the evolving intellectual landscape of schizophrenia research and point to emerging priorities such as personalized medicine and causal inference approaches. Importantly, this study demonstrates the value of bibliometric mapping tools such as VOSviewer in visualizing complex research structures and guiding strategic scientific inquiry. Future analyses could expand on these findings by incorporating additional databases, analyzing longitudinal topic evolution, or focusing on specific clinical‐genomic intersections in schizophrenia. Overall, this work provides a structured foundation for understanding research dynamics and informing future investigations in psychiatric genetics.

## Author Contributions

**Mohammad Narimani**: conceptualization, methodology, supervision, writing – review and editing. **Niloofar Mikaeili**: conceptualization, methodology, supervision, writing – review and editing. **Mahdi Naeim**: data curation, formal analysis, software, validation, visualization, writing – original draft.

## Funding

The authors have nothing to report.

## Ethics Statement

This study involved the analysis of publicly available bibliographic data from established academic databases (PubMed, scopus, and WoS) and did not include any research involving human participants, animals, or sensitive personal information. Therefore, ethical approval and informed consent were not required. All data were handled in accordance with the terms of use of the respective databases, ensuring proper citation and acknowledgment of original sources.

## Consent

This study is a bibliometric and scientometric analysis based on published literature. No individual participant data were collected, and therefore, no consent from participants was required.

## Conflicts of Interest

The authors declare no conflicts of interest.

## Data Availability

The original contributions presented in the study are included in the article, further inquiries can be directed to the corresponding author.
